# Routine frailty assessment predicts postoperative complications in elderly patients across surgical disciplines – a retrospective observational study

**DOI:** 10.1186/s12871-019-0880-x

**Published:** 2019-11-07

**Authors:** Oliver Birkelbach, Rudolf Mörgeli, Claudia Spies, Maria Olbert, Björn Weiss, Maximilian Brauner, Bruno Neuner, Roland C. E. Francis, Sascha Treskatsch, Felix Balzer

**Affiliations:** 1 Department of Anesthesiology and Operative Intensive Care Medicine (CCM, CVK), Charité – Universitätsmedizin Berlin, corporate member of Freie Universität Berlin, Humboldt-Universität zu Berlin, and Berlin Institute of Health, Charitéplatz 1, D-10117 Berlin, Germany; 2 Member of the Commission for Geriatric Anesthesiology of the German Society of Anesthesiology and Intensive Care Medicine (DGAI), Nuremberg, Germany

**Keywords:** Frailty, Elderly, Perioperative, Outcome

## Abstract

**Background:**

Frailty is a frequent and underdiagnosed functional syndrome involving reduced physiological reserves and an increased vulnerability against stressors, with severe individual and socioeconomic consequences. A routine frailty assessment was implemented at our preoperative anaesthesia clinic to identify patients at risk.

**Objective:**

This study examines the relationship between frailty status and the incidence of in-hospital postoperative complications in elderly surgical patients across several surgical disciplines.

**Design:**

Retrospective observational analysis.

**Setting:**

Single center, major tertiary care university hospital. Data collection took place between June 2016 and March 2017.

**Patients:**

Patients 65 years old or older were evaluated for frailty using Fried’s 5-point frailty assessment prior to elective non-cardiac surgery. Patients were classified into non-frail (0 criteria, reference group), pre-frail (1–2 positive criteria) and frail (3–5 positive criteria) groups.

**Main outcome measures:**

The incidence of postoperative complications was assessed until discharge from the hospital, using the roster from the National VA Surgical Quality Improvement Program. Propensity score matching and logistic regression analysis were performed.

**Results:**

From 1186 elderly patients, 46.9% were classified as pre-frail (*n* = 556), and 11.4% as frail (*n* = 135). The rate of complications were significantly higher in the pre-frail (34.7%) and frail groups (47.4%), as compared to the non-frail group (27.5%). Similarly, length of stay (non-frail: 5.0 [3.0;7.0], pre-frail: 7.0 [3.0;9.0], frail 8.0 [4.5;12.0]; *p* < 0.001) and discharges to care facilities (non-frail:1.6%, pre-frail: 7.4%, frail: 17.8%); p < 0.001) were significantly associated with frailty status. After propensity score matching and logistic regression analysis, the risk for developing postoperative complications was approximately two-fold for pre-frail (OR 1.78; 95% CI 1.04–3.05) and frail (OR 2.08; 95% CI 1.21–3.60) patients.

**Conclusions:**

The preoperative frailty assessment of elderly patients identified pre-frail and frail subgroups to have the highest rate of postoperative complications, regardless of age, surgical discipline, and surgical risk. Significantly increased length of hospitalisation and discharges to care facilities were also observed. Implementation of routine frailty assessments appear to be an effective tool in identifying patients with increased risk. Now future studies are needed to investigate whether patients benefit from optimization of patient counselling, process planning, and risk reduction protocols based on the application of risk stratification.

## Introduction

The concept of frailty and its relevance in the perioperative setting has been increasingly recognized in recent years [1–5]. Frailty describes a state of reduced physiological reserves, and a limited ability to compensate and recover from stressors. Surgery is often a major stressor, and current preoperative evaluation methods still fail to properly estimate physiological reserves [6]. The routine implementation of a frailty assessment could provide a more comprehensive and individualized perioperative risk stratification [3].

Although there is no commonly accepted definition of frailty, Fried’s description of “phenotypic frailty” is the most widely cited characterization of the syndrome, and was therefore selected for this assessment – for details see Table 1 [7]. Frailty can affect any age group, but it is more commonly found in older individuals, in combination with comorbidities and functional decline. In North America, approximately half of all surgical procedures are performed on patients aged 65 or older [8], and approximately 10% of this entire age segment is estimated to the frail [9]. Frail individuals are more likely to require surgery than their robust peers, and although assessments and populations vary considerably, 26–56% of all elderly surgical patients are reported to be frail [1]. As the population ages, the prevalence of frailty in the perioperative setting is also expected to rise.

**Table 1:** Frailty assessement

Frailty Criteria	Description	
Shrinking: weight loss	Unintentional weight loss ≥5 kg within the previous year	
Weakness: reduced grip strength (dominant hand), by gender and body mass index (BMI)	*Male*	*Female*
	BMI ≤24: ≤29 kg	BMI ≤23: ≤17 kg
	BMI 24.1-26: ≤30 kg	BMI 23.1-26: ≤17,3 kg
	BMI 26.1-28: ≤30 kg	BMI 26.1-29: ≤18 kg
	BMI >28: ≤32 kg	BMI >29: ≤21 kg
Exhaustion: answering C or D to the following question	How often in the past week did the following apply: “I felt that everything I did was an effort.” “I could not get going.”	
	a) Never or rarely	
	b) Sometimes	
	c) Often	
	d) Most of the time	
Gait Speed: slow walking speed (15 ft. = 4,57 m), dynamic start, by gender and height	*Male*	*Female*
	Height ≤ 173 cm: ≥ 7 s	Height ≤ 159 cm: ≥ 7 s
	Height > 173 cm: ≥ 6 s	Height > 159 cm: ≥ 6 s
Low activity	Metabolic Equivalent Tasks < 3	
Number of positive criteria	Frail: ≥3 criteria Intermediate / pre-frail: 1–2 criteria	

Frailty criteria utilized in the analysis, adapted from Fried [7]; *BMI* Body Mass Index

Frailty not only affects mortality rates, but is also associated with higher rates of complications and institutionalization, underlining the threat of lasting physical and cognitive disability following surgery [8, 10, 11]. An accurate risk stratification is thus crucial for healthcare providers and their patients prior to surgery. As part of a patient-oriented care, it is important to provide patients with realistic and individual information regarding their perioperative risk, recovery process, and long-term outcome. Since routine frailty assessments are poorly implemented, frail patients often undergo standard care without appropriate attention or preparation, erroneously expecting the same rate of recovery and functional improvement as their non-frail peers. Overall, frailty can have a severe impact on individual autonomy and quality of life, as well as significant socioeconomic consequences.

Most studies investigating the relationship between preoperative frailty and postoperative outcome only assess frailty retrospectively [12–14], indirectly estimating crucial frailty criteria, such as weakness and exhaustion. Evidence is still lacking as to whether patients benefit from a routine frailty assessment followed by an individualized treatment plan.

Therefore, the aim of this analysis is to examine the association between frailty (determined with a preoperative routine assessment) and the rate of in-hospital postoperative complications in elderly patients undergoing elective surgery. This analysis is to be understood as a preliminary work for follow-up studies that will investigate whether patients benefit from preoperative routine risk stratification.

## Methods

This retrospective cohort analysis examines data collected at the Campus Charité Mitte of the Charité – Universitätsmedizin Berlin, Germany, between June 2016 and March 2017. As part of routine pre-surgical assessment, patients undergoing elective surgery were seen at the anaesthesia preoperative clinic of the Department of Anaesthesiology and Intensive Care Medicine. The analysis was approved by the ethical committee (EA1/227/16) of the Charité Universitätsmedizin – Berlin, Berlin, Germany (Chairperson Prof. R. Uebelhack), on August 8th, 2016. Due to the retrospective nature, the requirement for written informed consent was waived by the ethics committee. The trial has been registered retrospectively at ClinicalTrials.gov (NCT03382054).

During the implementation period of this routine assessment, patients ≥65 years of age were offered a frailty assessment either at the preoperative anaesthesia clinic or on the peripheral wards. Surgical disciplines involved included general/gastrointestinal, orthopaedic, oral and maxillofacial surgery, as well as urology, gynaecology, otorhinolaryngology, and dermatology. This analysis does not include patients with emergency procedures or procedures without anaesthesia contribution or operation. Patients unable, unwilling, or unavailable to undergo the frailty assessment (patient refusal, language barrier, insufficient data, patient not found in room or unavailable due to other tests/examinations) were not recorded. Patients with multiple assessments, cancelled operation or cardiac surgery were excluded. Ultimately, one additional medical assistant position was required to establish a routine frailty assessment. This assistant, as well as two nurses from the preoperative anaesthesia clinic (as substitutes during vacation or illness), were trained in the frailty assessment (see Table 1) by a senior physician-scientist responsible for quality management (OB). The first several assessments were performed under supervision by the trainer, so as to corroborate understanding and quality. Training for the 5-point frailty criteria was deemed simple and required little training. The screening was done electronically via our hospital program, where all patients requiring anaesthesia must be registered. The assistant screened registered patients for inclusion criteria, and assessed eligible patients visiting the preoperative anaesthesia clinic prior to the visit with the physician. Patients were taken by the assistant to a designated room, which included the necessary equipment and dimensions for the frailty assessment (i.e. paper-based questionnaire, hand dynamometer, stopwatch, and >  5 m available for walking, with appropriate markings on the floor). The results were placed in the patient file and the patients returned to the waiting room. This assistant was also responsible for visiting the peripheral wards to assess the patients not visiting the clinic. After noting the name, station and room number of a registered patient, the assistant would take the necessary equipment in a “frailty bag”, which included the aforementioned equipment as well as measuring tape and small cones to mark distances. The assessment took place at the bedside and the walking test at the nearest hallway. After the assessment, the results were placed in the patient file and the assistant returned to the station. If no eligible patients were present, this assistant supported the remaining staff with the normal preoperative clinic program. Overall, the equipment required was durable and inexpensive. The workload was deemed low, with an average frailty assessment time of under 10 min and an average of 7–8 eligible patients per day.

General patient information was gathered, including age, sex, height, weight, smoking status, polypharmacy (routine intake of > 5 medication), American Society of Anesthesiologists Physical Status (ASA PS) classification, as well as comorbidities assessed by the Charlson Comorbidity Index (CCI) [15], surgical discipline, and preoperative creatinine levels. The surgical risk was classified according to European Society of Cardiology (ESC)/European Society of Anaesthesiology (ESA) guidelines on non-cardiac surgery into low, medium, or high risk [16]. Diagnoses for the entire hospitalization period and comorbidities were derived from our hospital database according to the International Statistical Classification of Diseases and Related Health Problems (ICD-10).

For the analysis, patients were classified into three groups according to the number of preoperative pathological frailty criteria described by Fried (0–5 criteria, see Table 1), consisting of non-frail (0 criteria, reference group), pre-frail (1–2 positive criteria), and frail (3–5 positive criteria) groups. Slight modifications were made to Fried’s frailty assessment in an attempt to adapt and improve data collection according to European standards, as summarized in a previous publication [17]. This included estimating weight loss in kilograms instead of pounds, and using a cut-off of ≥5 kg instead of ≥10 pounds (ca. 4.5 kg). In addition, metabolic equivalent tasks (METs) [18] were used instead of kilocalories/week (kCal/w). According to Fried, it is important to classify physical activity into low, moderate, and high levels, whereas a low level of activity in kCal/w is cited as a pathological criterion [7]. METs offer a different unit to evaluate physical activity, can also be classified into low, moderate, and high levels, and have the advantage of being faster and easier to use in clinical practice. Fried has defined physical activity in terms of METs [19], whereas a MET under 3 was considered low (and therefore pathological). As suggested by Fried, patients with > 2 missing criteria were removed from the analysis [7].

The primary outcome was the incidence and type of postoperative complications, which was selected in accordance with the Veteran Affairs’ National Surgical Quality Improvement Program (NSQIP) [20, 21] for purposes of comparability. Their standardized list of complications included pneumonia, pulmonary embolism, acute kidney injury, cerebrovascular accident, coma, superficial and deep wound surgical site infections, urinary tract infection, sepsis, deep vein thrombosis, reoperation, and reintubation due to respiratory/cardiac failure, myocardial infarction, cardiac arrest, and death. Although frailty assessments were performed by a trained staff assistant, outcome parameters were documented by healthcare documentation specialists into the hospital databank, who were not affiliated with his study. The hospital diagnoses were examined retrospectively by the authors for the presence or absence of ICD-10 codes corresponding to the NSQIP complications.

Although the frailty status of the patients were documented in the physical patient file, it was not noted in the electronic file nor the premedication records due to a missing interface, and no specific recommendations were made for the treatment of frail patients (minimizing performance bias). Outcome parameters were obtained from our hospital database, which were neither assessed nor documented by the frailty screening staff (minimizing measurement bias).

The evaluation of the data was carried out in an explorative approach. All data collected during the implementation period of the routine assessment (between June 2016 and March 2017) were available and were analysed considering the exclusion criteria. Due to the retrospective nature of this analysis, a sample size calculation was performed post-hoc, showing that 788 patients would be required to evaluate a difference between two groups (healthy vs pre-frail/frail) with a confidence of 80 and 5% alpha. Descriptive analyses and statistical testing were performed using the R Project of Statistical Computing, version 3.3.1. When normal distributions were ruled out using the Kolmogorov-Smirnov test, results were given as medians and interquartile ranges (IQR), otherwise as mean ± standard deviation (SD). Binary and ordinal variables were expressed by numbers with percentages. Differences in binary and ordinal variables between two independent groups were analysed by the exact chi-square test. In metric, non-normally distributed variables, differences between two independent groups were assessed with the Mann-Whitney-U-test and in ≥3 independent groups using the Kruskal-Wallis test. In metric, normally distributed variables, differences between groups were assessed using Student’s t-tests.

We removed the effect of baseline confounder variables by pairwise next neighbour matching (1:1:1). This includes a propensity score creation and next neighbour matching for the first and second group, followed by an additional propensity score creation and matching for the second and third groups, with group order 0 (non-frail), 1 (pre-frail) and 2 (frail). Propensity score matching was performed using the R package “MatchIt” version 3.0.2, based on Ho et al. [22]. The following baseline characteristics were included, as there were considered to be major confounders: age, sex, body mass index, ASA PS, surgical risk, type of anaesthesia, CCI, surgical discipline, smoking status, polypharmacy, as well as preoperative creatinine levels and glomerular filtration rates (GFR) as surrogates for chronic kidney injury. Additionally, the following comorbidities were also included: coronary artery disease, peripheral artery disease, diabetes mellitus, liver disease, tumour, cardiac failure, cerebrovascular accident, asthma bronchiale, and chronic obstructive pulmonary disease. Baseline characteristics that remained statistically significant after propensity score matching were included in a subsequent logistic regression model with frailty status as further explanatory variable. Since propensity score matching presents a method of regression analysis itself, subjecting variables that have already been successfully controlled in propensity score matching (i.e. *p* < 0.05) to a subsequent logistic regression would not improve the analysis. The regression’s target variable was compound complications. A two-tailed *p*-value < 0.05 was considered statistically significant. All tests should be understood as constituting explorative analysis; no adjustment for multiple testing has been made.

## Results

**Fig. 1:**
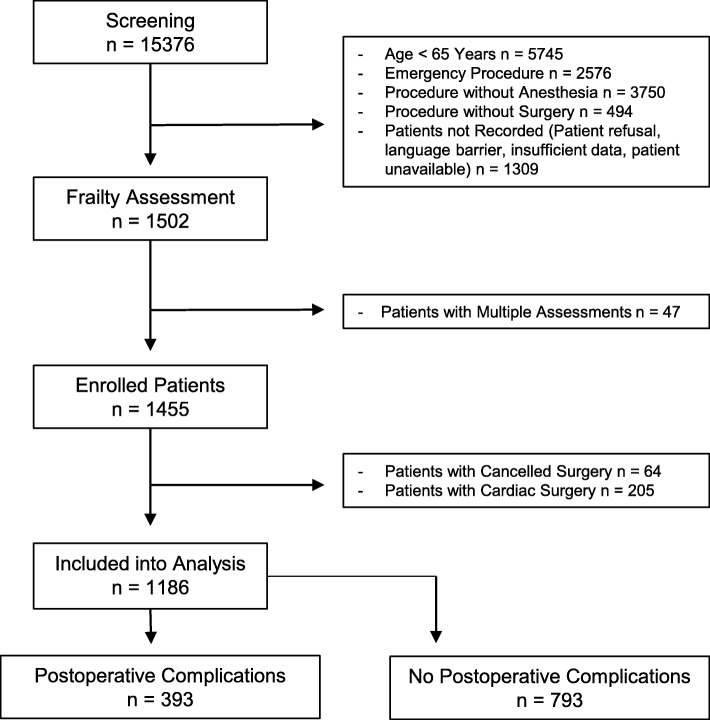
Flow Chart

A total of 1186 patients were included in the analysis (for details see Fig. 1). Patient characteristics, common comorbidities, and distribution across surgical disciplines are described in Table 2.

**Table 2:** Patient Characteristics

Patient Characteristics	*N* = 1186
Age	74.0 [70.0;78.0]
Male	623 (52.5%)
BMI	26.2 [23.6;29.4]
ASA Score ≥ 3	505 (42.6%)
ESC/ESA Surgical Risk	
High	29 (2.45%)
Intermediate	778 (65.6%)
Low	379 (32.0%)
General anesthesia	1106 (93.3%)
CCI	2.0 [1.0;5.0]
Surgical Discipline:	
Orthopedic	384 (32.4%)
Urology	265 (22.3%)
Otorhinolaryngology	202 (17.0%)
General/Visceral	192 (16.2%)
Gynecology	98 (8.3%)
Others	45 (3.8%)
Smoking-Status:	
Yes, active	165 (14.1%)
No, quit	412 (35.3%)
No, never	591 (50.6%)
Polypharmacy (> 5 drugs)	541 (46.1%)
Preop Creatinine (mg/dL)	0.92 [0.77;1.11]
GFR (MDRD)	75.9 [62.1;88.6]
Pre-existing conditions:	
CAD	201 (16.9%)
PAD	141 (11.9%)
Diabetes mellitus	217 (18.3%)
Liver Disease	52 (4.4%)
Tumor	537 (45.3%)
Cardiac Failure	145 (12.2%)
Cerebrovascular Accident	103 (8.7%)
Asthma/COPD	266 (22.4%)

*BMI* Body Mass Index, *ASA PS* American Society of Anesthesiologists Physical Status, *ESC* European Society of Cardiology, *ESA* European Society of Anaesthesiology, *CCI* Charlson Comorbidity Index, *GFR (MDRD)* Glomerular filtration rate (Modification of Diet in Renal Disease study equation), *CAD* Coronary artery disease, *PAD* Peripheral artery disease, *COPD* Chronic obstructive pulmonary disease

**Fig. 2:**
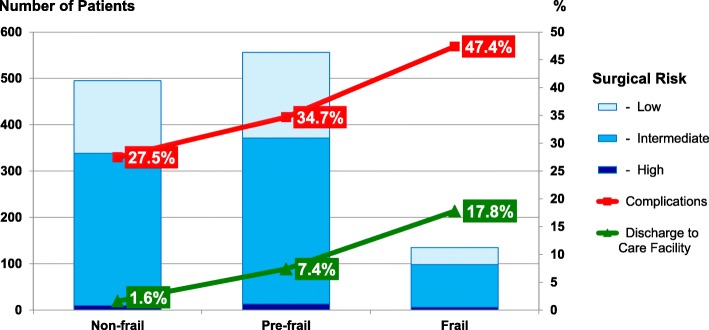
Incidence of postoperative complications and discharge to care facility by frailty status and according to surgical risk

Overall, 556 patients (46.9%) were found to be pre-frail, and 135 (11.4%) frail. Table 3 shows the incidence of (NSQIP) in-hospital postoperative complications across all groups, including ICD-10 codes. One or more complications were observed in 393 cases (33.1%), whereas the incidence of postoperative complications were strongly associated with the presence of frailty characteristics (*p* < 0.01, see Fig. 2). Additionally, length of stay and discharge to care facilities were also strongly associated with frailty status (both p < 0.01, see Table 3).

**Table 3:** Complication rates by frailty status

Complication Rates	ICD-10	All (*n* = 1186)	Non-Frail (*n* = 495)	Pre-Frail (*n* = 556)	Frail (*n* = 135)	*p*-Value
Cardiac Arrest	I46	7 (0.6%)	0 (0.0%)	3 (0.5%)	4 (3.0%)	0.001
Cardiac Infarct	I21	4 (0.3%)	0 (0.0%)	2 (0.4%)	2 (1.5%)	0.036
Pneumonia	J13-J18, J20-J22	28 (2.36%)	7 (1.41%)	16 (2.88%)	5 (3.70%)	0.143
Pulmonary Embolism	I26	6 (0.5%)	0 (0.0%)	6 (1.1%)	0 (0.0%)	0.062
Acute Kidney Injury	N17, N19	69 (5.82%)	19 (3.84%)	38 (6.83%)	12 (8.89%)	0.032
Cerebrovascular Accident	I61-I64	3 (0.3%)	1 (0.2%)	1 (0.2%)	1 (0.7%)	0.479
Coma	R40	4 (0.3%)	1 (0.2%)	1 (0.2%)	2 (1.5%)	0.096
Deep Wound Infection	T81.3	18 (1.5%)	3 (0.6%)	12 (2.2%)	3 (2.2%)	0.061
Superficial Wound Infection	T81.4	34 (2.9%)	6 (1.2%)	20 (3.6%)	8 (5.9%)	0.004
Urinary Tract Infection	N30, N32-N34, N39	205 (17.3%)	75 (15.2%)	97 (17.4%)	33 (24.4%)	0.040
Sepsis	A40-A41	18 (1.5%)	2 (0.4%)	12 (2.2%)	4 (3.0%)	0.011
Deep Vein Thrombosis	I80-I82	14 (1.18%)	3 (0.61%)	9 (1.62%)	2 (1.48%)	0.243
Re-operation		117 (9.9%)	39 (7.9%)	59 (10.6%)	19 (14.1%)	0.073
Re-intubation		31 (2.6%)	7 (1.4%)	18 (3.2%)	6 (4.4%)	0.049
Complications (Total)		393 (33.1%)	136 (27.5%)	193 (34.7%)	64 (47.4%)	< 0.001
Length of Stay (days)		6.0 [3.0;9.0]	5.0 [3.0;7.0]	7.0 [3.0;9.0]	8.0 [4.5;12.0]	< 0.001
Discharge to Care Facility		73 (6.2%)	8 (1.6%)	41 (7.4%)	24 (17.8%)	< 0.001

Total shown as number of patients with at least one complication. National Surgical Quality Improvement Program complication according to [20, 21] and their frequencies by frailty status. ICD-10: International Statistical Classification of Diseases and Related Health Problems

Non-frail and pre-frail patients were matched with the frail group according to sex, BMI, ASA PS, ESC/ESA surgical risk, CCI, smoking status, surgical discipline, and comorbidities. After matching, significant differences were observed in respect to age (*p* < 0.001), polypharmacy (*p* < 0.001), and history of cardiac failure (*p* = 0.003), as shown in Table 4. Therefore, these variables were included in a subsequent logistic regression analysis (see Table 5). Pre-frail patients were shown to be nearly 1.8 times more likely to develop complications than their non-frail peers (OR 1.778; 95% CI 1.043–3.052), whereas frail patients had a 2-fold increase in risk (OR 2.078; 95% CI 1.212–3.596). In this model, age and history of heart failure were no longer independent predictors of statistical significance. Polypharmacy was associated with a 1.6 increase for developing complications (OR 1.633; 95% CI 1.017 to 2.648).

**Table 4:** Propensity score matching

Matching Criteria	Non-Frail (*n* = 135)	Pre-Frail (*n* = 135)	Frail (*n* = 135)	*p*-Value
Age	75.0 [71.5;78.0]	77.0 [74.0;82.0]	77.0 [73.5;80.0]	< 0.001
Male	70 (51.9%)	64 (47.4%)	65 (48.1%)	0.736
BMI	26.0 [23.1;29.1]	26.2 [23.4;30.2]	26.6 [23.0;30.3]	0.725
ASA Score ≥ 3	90 (66.7%)	105 (77.8%)	102 (75.6%)	0.092
ESC/ESA Surgical Risk				0.221
High	3 (2.2%)	5 (3.7%)	6 (4.4%)	
Intermediate	79 (58.5%)	91 (67.4%)	92 (68.1%)	
Low	53 (39.3%)	39 (28.9%)	37 (27.4%)	
General anesthesia	124 (91.9%)	121 (89.6%)	125 (92.6%)	0.666
CCI	3.0 [1.0;6.0]	3.0 [1.0;6.0]	4.0 [2.0;6.5]	0.142
Surgical Discipline:				0.235
Orthopedic	48 (35.6%)	65 (48.1%)	62 (45.9%)	
Urology	33 (24.4%)	27 (20.0%)	22 (16.3%)	
Otorhinolaryngology	12 (8.9%)	15 (11.1%)	21 (15.6%)	
General/Visceral	26 (19.3%)	18 (13.3%)	22 (16.3%)	
Gynecology	10 (7.4%)	8 (5.9%)	5 (3.7%)	
Others	6 (4.4%)	2 (1.5%)	3 (2.2%)	
Smoking-Status:				0.641
Yes, active	21 (15.7%)	15 (11.4%)	22 (16.4%)	
No, quit	54 (40.3%)	49 (37.1%)	48 (35.8%)	
No, never	59 (44.0%)	68 (51.5%)	64 (47.8%)	
Polypharmacy (> 5 drugs)	66 (49.3%)	88 (66.2%)	111 (83.5%)	< 0.001
Preop Creatinine (mg/dL)	0.93 [0.79;1.16]	0.96 [0.78;1.16]	0.94 [0.79;1.23]	0.663
GFR (MDRD)	74.7 [59.1;86.3]	69.8 [53.7;81.5]	68.8 [50.8;87.7]	0.302
Pre-existing Conditions:				
CAD	33 (24.4%)	36 (26.7%)	37 (27.4%)	0.847
PAD	22 (16.3%)	26 (19.3%)	28 (20.7%)	0.635
Diabetes mellitus	33 (24.4%)	40 (29.6%)	43 (31.9%)	0.385
Liver Disease	8 (5.9%)	8 (5.9%)	13 (9.6%)	0.395
Tumor	64 (47.4%)	57 (42.2%)	53 (39.3%)	0.392
Cardiac Failure	18 (13.3%)	37 (27.4%)	40 (29.6%)	0.003
Cerebrovascular Accident	10 (7.4%)	17 (12.6%)	18 (13.3%)	0.241
Asthma/COPD	45 (33.3%)	49 (36.3%)	48 (35.6%)	0.869
Complications (Total)	36 (26.7%)	55 (40.7%)	64 (47.4%)	0.002
Length of Stay (days)	5.0 [3.0;8.0]	8.0 [3.0;10.5]	8.0 [4.5;12.0]	< 0.001
Discharge to Care Facility	2 (1.48%)	15 (11.1%)	24 (17.8%)	< 0.001

Total shown as number of patients with at least one complication. *BMI* Body Mass Index; *ASA PS* American Society of Anesthesiologists Physical Status, *ESC* European Society of Cardiology, *ESA* European Society of Anaesthesiology, *CCI* Charlson Comorbidity Index, *GFR (MDRD)* Glomerular filtration rate (Modification of Diet in Renal Disease study equation), *CAD* Coronary artery disease, *PAD* Peripheral artery disease, *COPD* Chronic obstructive pulmonary disease

**Table 5:** Logistic regression results

Factor	*P*-Values	OR	95% CI
Non-Frailty	Ref.	Ref.	Ref.
Pre-Frailty	0.035	1.778	1.043 to 3.052
Frailty	0.008	2.078	1.212 to 3.596
Age	0.898	0.998	0.960 to 1.036
Polypharmacy	0.044	1.633	1.017 to 2.648
History of Cardiac Failure	0.178	1.402	0.856 to 2.291

Model contains all remaining significant factors from Propensity Score Matching (see Table 4). *OR* Odds Ratio, *CI* Confidence Interval

## Discussion

The aim of this study was to analyse the relationship between preoperative frailty and the incidence of postoperative complications in elderly patients undergoing a wide range of non-cardiac elective surgery in a major European tertiary care university hospital. The analysis was based on a large-scale routine frailty assessment for patients 65 years of age or older. Overall, 58.3% of surgical patients were found to be either pre-frail or frail, subsequently showing an increased incidence of postoperative complications. In our analysis, phenotypic pre-frailty and frailty were strongly associated with an increased risk for postoperative complications, increased length of hospitalisation, and risk of discharge to care facilities in elderly patients among a wide variety of disciplines and surgical interventions.

In order to gain some insight into the relevance of frailty’s physical aspects, we performed a propensity score matching. Unsurprisingly, age remained a statistically significant factor for complications in the matched groups, as the accumulation of comorbidities and functional decline correlate with age. When adjusting for other significant variables (pre-frailty, frailty, polypharmacy, and history of cardiac failure), age ceased to be an independent predictor for postoperative complications. This is in line with the work of Suskind and colleagues [23] on urological interventions, which found frailty to be an independent predictor of postoperative complications, irrespective of age, up to octogenarians. In our analysis, pre-frailty and frailty, as well as polypharmacy, remained significant predictors of in-hospital postoperative complications.

Our results are further supported by a number of smaller investigations [6, 8, 10, 24–27], prospective and retrospective, which used a variety of tools to suggest an association between frailty and postoperative outcomes in specific surgical populations. Makary and colleagues [8] assessed 594 patients using Fried’s criteria, examining their predictive power in the postoperative context in combination with risk indices. Revenig and colleagues [6] described 80 patients, ranging from 19 to 87 years old, undergoing minimally invasive surgery, and described a higher rate of complications in frail patients (Fried’s phenotype). Robinson [21] reported higher rates of postoperative complications in 201 frail elderly patients undergoing colorectal and cardiac surgery, while using their own 7-point frailty scale. Dasgupta [25] assessed 125 elderly patients using the Edmonton Frail Scale to find a higher complication rate after non-cardiac elective surgery (85% of which were orthopaedic interventions).

Fried’s phenotype assessment is the most often cited method for determining frailty [28], but other domains should certainly be considered (e.g. cognitive, psychosocial aspects). The next most cited frailty assessment is the Deficit Accumulation Model from Rockwood [29], which does not focus on physical aspects, but rather encompasses several frailty domains. However, Rockwood’s test is comprised of a significantly larger test battery, which is more demanding on terms of training, equipment, and resources. The choice to implement Fried’s phenotype was made under consideration that a larger pool of publications would enhance the study’s background and allow broader comparability, in addition to a modest resource requirement for implementation.

In a previous article, we summarized a frailty assessment based on the Fried criteria that seems feasible in preoperative routine care and at the same time adequately describes the phenotype [17]. This study indicates that a frailty assessment is practicable in a routine setting, and is able to identify patients at higher risk for complications. Although workload will vary significantly, implementation of routine assessment should be critically considered by other clinics, especially in light of its potential to improve perioperative pathways.

The high degree of variability among the above-mentioned studies in terms of frailty definition, patient population, and outcome measures, possibly delays implementation in the clinical routine, thus calling for a large-scale analysis on the predictive value of frailty assessment across surgical disciplines. With this analysis, we provide data for the first time from a large European cohort with routine frailty assessment investigating the impact of frailty across several surgical disciplines.

Ideally, frail patients should undergo an individualized perioperative pathway, including an interdisciplinary shared decision-making conference to ascertain deficits, risks, and therapy goals. Additionally, prehabilitation measures may also be employed in an attempt to improve preoperative status and minimize perioperative risk. These are current objects of research and require considerable resources. However, identifying frail individuals and recognizing them as high-risk patients remain the primary step for the deployment of risk reduction strategies. Regardless of interdisciplinary conferences or prehabilitation programs, simply being aware of a patient’s frailty status allows us to implement perioperative preventive measures and heighten our vigilance for complications in this vulnerable collective. These measures may be employed before, during, or after the operation, and include steps such as preoperative warming, careful choice of anaesthetics, advanced hemodynamic and neuromonitoring, appropriate delirium and pain management, early mobilization, and others [30].

There are a number of limitations in this investigation that must be considered. Screening was offered to all patients undergoing elective surgery, whether seen at the preoperative anaesthesia clinic or at the peripheral wards. However, a selection bias may nevertheless be present, as patients in the periphery were more often not found in their rooms or were unavailable due to other tests or examinations, and many could not be revisited prior to the operation. Due to the large range of surgical interventions, the influence of type and duration of surgery was not included in the analysis. The NSQIP list of complications was selected for this study, as it offered a standard for comparability with similar studies, however, this decision did limit the scope of complications analysed. Outcome parameters were not rated into minor/major categories, and were derived from ICD-10 coded hospital diagnoses, so that limitations of routine data use are applicable. The decision to employ a propensity score focused our analysis on physical aspects of frailty, while neutralizing a number of other frailty domains that may also impact patient outcome. Although a surprisingly high rate of urinary tract infections was observed in this study, a sub-analysis ignoring this complication showed no significant difference in the results. Due to the retrospective nature of the analysis and waived informed consent, follow-up attempts on out-of-hospital complications, re-admission rates, or death following discharge could not be performed. Our hospital has implemented postoperative management concepts aimed at reducing complications, namely the modified Hospital Elder Life Program (mHELP) [31] and Enhanced Recovery After Surgery (ERAS) [32], which may have had an effect on the observed complication rates. These programs were well-established and no changes in the protocol of either program took place during the study period. Lastly, the analysis present data of a single centre academic hospital, and a multicentre evaluation, including major and minor medical centres, might provide more generalizable evidence.

Further studies are required to determine specific risk factors, as well as the impact of other frailty dimensions (e.g. cognitive impairment, social frailty). Lastly, large-scale projects are needed to develop and analyse potential interventions that may limit the effects of frailty in surgical populations.

In conclusion, we present evidence that the Fried’s frailty phenotype assessment is a clinically relevant predictor for in-hospital postoperative complications across a variety of surgical specialties, and can be easily implemented in clinical routine. Pre-frailty and frailty, independent of age, can identify patients at risk, and may be used to optimize patient counselling, process planning, and risk reduction protocols.

## Data Availability

The datasets used and analysed during the current study are available from the corresponding author on reasonable request.

## Abbreviations

ASA PS: American Society of Anesthesiologists Physical Status
BMI: Body mass index
CAD: Coronary artery disease
CCI: Charlson comorbidity index
CI: Confidence interval
COPD: Chronic obstructive pulmonary disease
ERAS: Enhanced recovery after surgery
ESA: European society of anaesthesiology
ESC: European society of cardiology
ICD-10: International Statistical Classification of Diseases and Related Health Problems
IQR: Interquartile Ranges
kCal/w: Kilocalories/Week
METs: Metabolic Equivalent Tasks
mHELP: Hospital Elder Life Program
NSQIP: Veteran Affairs’ National Surgical Quality Improvement Program
OR: Odds ratio
PAD: Peripheral artery disease
SD: Standard deviation

## References

[1] Amrock LG, Deiner S (2014). The implication of frailty on preoperative risk assessment. Curr Opin Anaesthesiol.

[2] Anaya DA, Johanning J, Spector SA, Katlic MR, Perrino AC, Feinleib J (2014). Summary of the panel session at the 38th annual surgical symposium of the association of VA surgeons: what is the big deal about frailty?. JAMA Surg..

[3] Buigues C, Juarros-Folgado P, Fernández-Garrido J, Navarro-Martínez R, Cauli O (2015). Frailty syndrome and pre-operative risk evaluation: a systematic review. Arch Gerontol Geriatr.

[4] Lin H-S, Watts JN, Peel NM, Hubbard RE (2016). Frailty and post-operative outcomes in older surgical patients: a systematic review. BMC Geriatr.

[5] Shem Tov L, Matot I. Frailty and anesthesia. Curr Opin Anaesthesiol. 2017. 10.1097/ACO.0000000000000456 28291129

[6] Revenig LM, Canter DJ, Master VA, Maithel SK, Kooby DA, Pattaras JG (2014). A prospective study examining the association between preoperative frailty and postoperative complications in patients undergoing minimally invasive surgery. J Endourol.

[7] Fried LP, Tangen CM, Walston J, Newman AB, Hirsch C, Gottdiener J (2001). Frailty in older adults: evidence for a phenotype. J Gerontol A Biol Sci Med Sci.

[8] Makary MA, Segev DL, Pronovost PJ, Syin D, Bandeen-Roche K, Patel P (2010). Frailty as a predictor of surgical outcomes in older patients. J Am Coll Surg.

[9] Collard RM, Boter H, Schoevers RA, Oude Voshaar RC (2012). Prevalence of frailty in community-dwelling older persons: a systematic review. J Am Geriatr Soc.

[10] Robinson TN, Wallace JI, Wu DS, Wiktor A, Pointer LF, Pfister SM (2011). Accumulated frailty characteristics predict postoperative discharge institutionalization in the geriatric patient. J Am Coll Surg.

[11] Hewitt J, Moug SJ, Middleton M, Chakrabarti M, Stechman MJ, McCarthy K (2015). Prevalence of frailty and its association with mortality in general surgery. Am J Surg.

[12] Traven SA, Reeves RA, Slone HS, Walton ZJ (2019). Frailty predicts medical complications, length of stay, readmission, and mortality in revision hip and knee Arthroplasty. J Arthroplast.

[13] Wahl TS, Graham LA, Hawn MT, Richman J, Hollis RH, Jones CE (2017). Association of the Modified Frailty Index with 30-day surgical readmission. JAMA Surg.

[14] Rothenberg KA, Stern JR, George EL, Trickey AW, Morris AM, Hall DE (2019). Association of Frailty and Postoperative Complications with Unplanned Readmissions after Elective Outpatient Surgery. JAMA Netw Open.

[15] Charlson M, Szatrowski TP, Peterson J, Gold J (1994). Validation of a combined comorbidity index. J Clin Epidemiol.

[16] Kristensen SD, Knuuti J, Saraste A, Anker S, Bøtker HE, De Hert S (2014). 2014 ESC/ESA guidelines on non-cardiac surgery: cardiovascular assessment and management: the joint task force on non-cardiac surgery: cardiovascular assessment and management of the European Society of Cardiology (ESC) and the European Society of Anaesthesiology (ESA). Eur J Anaesthesiol.

[17] Birkelbach O, Mörgeli R, Balzer F, Olbert M, Treskatsch S, Kiefmann R (2017). Why and how should I assess frailty? A guide for the preoperative anesthesia clinic. Anasthesiol Intensivmed Notfallmed Schmerzther.

[18] Liu N, Pruszkowski O, Leroy JE, Chazot T, Trillat B, Colchen A (2013). Automatic administration of propofol and remifentanil guided by the bispectral index during rigid bronchoscopic procedures: a randomized trial. Can J Anaesth.

[19] Siscovick DS, Fried L, Mittelmark M, Rutan G, Bild D, O'Leary DH (1997). Exercise intensity and subclinical cardiovascular disease in the elderly. The cardiovascular health study. Am J Epidemiol.

[20] Khuri SF, Daley J, Henderson W, Hur K, Demakis J, Aust JB (1998). The Department of Veterans Affairs' NSQIP: the first national, validated, outcome-based, risk-adjusted, and peer-controlled program for the measurement and enhancement of the quality of surgical care. National VA surgical quality improvement program. Ann Surg.

[21] Robinson TN, Wu DS, Pointer L, Dunn CL, Cleveland JC, Moss M (2013). Simple frailty score predicts postoperative complications across surgical specialties. Am J Surg.

[22] Ho D, Imai K, King G, Stuart EA. MatchIt: Nonparametric Preprocessing for Parametric Causal Inference. 2011. 2011;42(8):28.

[23] Suskind AM, Walter LC, Jin C, Boscardin J, Sen S, Cooperberg MR (2016). Impact of frailty on complications in patients undergoing common urological procedures: a study from the American College of Surgeons National Surgical Quality Improvement database. BJU Int.

[24] Kristjansson SR, Nesbakken A, Jordhøy MS, Skovlund E, Audisio RA, Johannessen H-O (2010). Comprehensive geriatric assessment can predict complications in elderly patients after elective surgery for colorectal cancer: a prospective observational cohort study. Crit Rev Oncol Hematol.

[25] Dasgupta M, Rolfson DB, Stolee P, Borrie MJ, Speechley M (2009). Frailty is associated with postoperative complications in older adults with medical problems. Arch Gerontol Geriatr.

[26] Saxton A, Velanovich V (2011). Preoperative frailty and quality of life as predictors of postoperative complications. Ann Surg.

[27] Lee DH, Buth KJ, Martin B-J, Yip AM, Hirsch GM (2010). Frail patients are at increased risk for mortality and prolonged institutional care after cardiac surgery. Circulation..

[28] Buta BJ, Walston JD, Godino JG, Park M, Kalyani RR, Xue Q-L (2016). Frailty assessment instruments: systematic characterization of the uses and contexts of highly-cited instruments. Ageing Res Rev.

[29] Rockwood K, Song X, MacKnight C, Bergman H, Hogan DB, McDowell I (2005). A global clinical measure of fitness and frailty in elderly people. CMAJ..

[30] Morgeli R, Wollersheim T, Spies C, Balzer F, Koch S, Treskatsch S (2017). How to reduce the rate of postoperative complications in frail patients?. Anästhesiol Intensivmed Notfallmed Schmerzther.

[31] Chen CC-H, Chen C-N, Lai IR, Huang G-H, Saczynski JS, Inouye SK (2014). Effects of a modified hospital elder life program on frailty in individuals undergoing major elective abdominal surgery. J Am Geriatr Soc.

[32] Feldheiser A, Aziz O, Baldini G, Cox BPBW, Fearon KCH, Feldman LS (2016). Enhanced recovery after surgery (ERAS) for gastrointestinal surgery, part 2: consensus statement for anaesthesia practice. Acta Anaesthesiol Scand.

